# Comparison of non-invasive assessment of liver fibrosis in patients with alpha1-antitrypsin deficiency using magnetic resonance elastography (MRE), acoustic radiation force impulse (ARFI) Quantification, and 2D-shear wave elastography (2D-SWE)

**DOI:** 10.1371/journal.pone.0196486

**Published:** 2018-04-26

**Authors:** Rolf Reiter, Martin Wetzel, Karim Hamesch, Pavel Strnad, Patrick Asbach, Matthias Haas, Britta Siegmund, Christian Trautwein, Bernd Hamm, Dieter Klatt, Jürgen Braun, Ingolf Sack, Heiko Tzschätzsch

**Affiliations:** 1 Department of Radiology, Charité - Universitätsmedizin Berlin, corporate member of Freie Universität Berlin, Humboldt-Universität zu Berlin, and Berlin Institute of Health, Berlin, Germany; 2 Richard and Loan Hill Department of Bioengineering, College of Medicine and College of Engineering, University of Illinois at Chicago, Chicago, Illinois, United States of America; 3 Medical Department, Division of Gastroenterology, Infectiology, and Rheumatology, Campus Benjamin Franklin, Charité - Universitätsmedizin Berlin, corporate member of Freie Universität Berlin, Humboldt-Universität zu Berlin, and Berlin Institute of Health, Berlin, Germany; 4 Medical Clinic III, Gastroenterology, Metabolic Diseases, and Intensive Care, University Hospital RWTH Aachen, Aachen, Germany; 5 Coordinating center for alpha1-antitrypsin deficiency-related liver disease of the European Reference Network (ERN) “Rare Liver” and the European Association for the Study of the Liver (EASL) registry group “Alpha1-Liver”, Aachen, Germany; 6 Institute of Medical Informatics, Charité - Universitätsmedizin Berlin, corporate member of Freie Universität Berlin, Humboldt-Universität zu Berlin, and Berlin Institute of Health, Berlin, Germany; Linköping University, SWEDEN

## Abstract

**Purpose:**

Although it has been known for decades that patients with alpha1-antitrypsin deficiency (AATD) have an increased risk of cirrhosis and hepatocellular carcinoma, limited data exist on non-invasive imaging-based methods for assessing liver fibrosis such as magnetic resonance elastography (MRE) and acoustic radiation force impulse (ARFI) quantification, and no data exist on 2D-shear wave elastography (2D-SWE). Therefore, the purpose of this study is to evaluate and compare the applicability of different elastography methods for the assessment of AATD-related liver fibrosis.

**Methods:**

Fifteen clinically asymptomatic AATD patients (11 homozygous PiZZ, 4 heterozygous PiMZ) and 16 matched healthy volunteers were examined using MRE and ARFI quantification. Additionally, patients were examined with 2D-SWE.

**Results:**

A high correlation is evident for the shear wave speed (SWS) determined with different elastography methods in AATD patients: 2D-SWE/MRE, ARFI quantification/2D-SWE, and ARFI quantification/MRE (*R* = 0.8587, 0.7425, and 0.6914, respectively; *P*≤0.0089). Four AATD patients with pathologically increased SWS were consistently identified with all three methods—MRE, ARFI quantification, and 2D-SWE.

**Conclusion:**

The high correlation and consistent identification of patients with pathologically increased SWS using MRE, ARFI quantification, and 2D-SWE suggest that elastography has the potential to become a suitable imaging tool for the assessment of AATD-related liver fibrosis. These promising results provide motivation for further investigation of non-invasive assessment of AATD-related liver fibrosis using elastography.

## 1. Introduction

Alpha1-antitrypsin deficiency (AATD) is a common, but frequently overlooked, autosomal co-dominant hereditary disorder affecting the lungs and liver. Besides pulmonary emphysema, AATD is known to cause chronic hepatitis, liver fibrosis, cirrhosis, and hepatocellular carcinoma (HCC). The two largest screening studies in newborns were performed in 107,038 infants in Oregon, USA, and in 200,000 infants in Sweden and found a prevalence of 1/5097 and 1/1639, respectively [1,2]. Stoller *et al*. estimate that less than 10% of the approximately 70,000 to 100,000 AATD patients in the United States have been diagnosed [3]. AATD-related cirrhosis and HCC are predominantly observed in male Caucasians from Europe and North America [4–6]. In a cohort of 647 AATD patients in Florida, USA, a prevalence of 7.9% of any liver disease and 3.4% of cirrhosis was observed [7]. An autopsy study, covering a 20-year period from 1963–1982, revealed cirrhosis in approximately 35–40% of adult homozygous AATD (PiZZ) patients at the time of death [6]. Most of these patients did not have any known liver disease. The same study reported a mean survival of 2 years after the diagnosis of cirrhosis with portal hypertension [6].

The alpha1-antitrypsin (AAT) molecule is a serine protease inhibitor that is mainly synthesized in the liver and secreted into the blood stream. One of its most important functions is to protect lung tissue against aggressive proteolytic enzymes such as neutrophil elastase, cathepsin G, and proteinase 3 [8]. In patients with the PiZZ genotype, glutamate is substituted for lysine at position 342 of the polypeptide chain in the AAT gene (SERPINA1). This mutation changes the tertiary structure and causes the formation of falsely folded glycoprotein conglomerates in the endoplasmic reticulum of hepatocytes [8]. An accumulation of insoluble AAT in hepatocytes leads to apoptosis and hepatic inflammation, which trigger fibrogenesis and hepatocarcinogenesis [9]. Patients with a heterozygous genotype (i.e., PiMZ) develop less severe clinical features. Overall, for reasons not yet known, there is wide variation in the development of liver disease [10].

Diagnostic tests include determination of AAT blood concentration and genotyping. In contrast to pulmonary involvement and other chronic liver diseases, the ongoing AATD-related damage of liver tissue is mostly a clinically silent process without elevated liver enzymes. Clark *et al*. reported that even in patients with AATD-related cirrhosis, no significant elevation of alanine transaminase was evident [7]. Because of this diagnostic challenge, liver involvement is frequently overlooked and discovered only once patients have developed cirrhosis or HCC, which is associated with an unfavorable prognosis [5]. Currently, there is no effective treatment for AATD-related liver disease except for liver transplantation.

Liver biopsy is the invasive gold standard for staging liver fibrosis. However, due to pain and the risk of complications, liver biopsy does not seem to be adequate for the screening or follow-up of asymptomatic patients. Additionally, the limited diagnostic accuracy of this invasive method has been shown by many studies [11–13].

Elastography is an emerging imaging technique for the non-invasive assessment of mechanical tissue properties [14–17]. Quantitative measurement using shear wave excitation of biological tissue is a valuable complement to qualitative morphological imaging modalities such as magnetic resonance imaging (MRI) and ultrasound (US). According to the 2017 version of the appropriateness criteria of chronic liver disease issued by the American College of Radiology, magnetic resonance elastography (MRE) is the most accurate non-invasive method for assessing liver fibrosis, allowing assessment of the entire liver even in patients with ascites and obesity [18]. Drawbacks are general MRI contraindications such as cardiac pacemakers or defibrillators, cochlear implants, hepatic iron overload, and claustrophobia. Ultrasound elastography (USE) techniques such as acoustic radiation force impulse (ARFI) quantification and 2D-shear wave elastography (2D-SWE) have several advantages: (i) higher availability, (ii) faster examination, (iii) lower costs, (iv) fewer contraindications, and (v) more established cut-off values for staging liver fibrosis [19–21]. Additionally, 2D-SWE has the benefit of a larger field of view compared to ARFI quantification.

Although it has been known for decades that patients with AATD have an increased risk of cirrhosis and HCC, limited data exist on non-invasive imaging-based methods for assessing liver fibrosis such as MRE and ARFI quantification, and no data exist on 2D-SWE. Therefore, the purpose of this study is to evaluate and compare the applicability of different elastography methods for the assessment of AATD-related liver fibrosis.

## 2. Patients and methods

In this prospective study, 15 AATD patients (11 PiZZ, 4 PiMZ) and 16 healthy volunteers were examined using MRE and ARFI quantification. Additionally, patients were examined with 2D-SWE. Healthy volunteers were matched for age, sex, and body mass index (BMI). The study was approved by the institutional review board (ethics committee) of the Charité - Universitätsmedizin Berlin, Germany, and written informed consent was obtained from all participants. AATD was confirmed by measurement of AAT serum levels, and genotyping if indicated. Inclusion criteria were AATD patients of all genotypes and age of 18 to 79 years. Exclusion criteria were decompensated cirrhosis, viral hepatitis, acute hepatopathies, cholestasis, signs of right heart failure, increased daily alcohol consumption (>30 g/d for men, >20 g/d for women), malignant disease, pregnancy, and general MRI contraindications. Before USE and MRE, all participants had fasted for at least 4 h to avoid an increased postprandial liver stiffness [22]. The examinations were performed by three experienced physicians with 3 (K.M.), 5 (M.W.), and 7 (R.R.) years of experience in elastography imaging. ARFI quantification and 2D-SWE were performed on the same day, while MRE followed after a median of 125 days (interquartile range: 28 days). In healthy volunteers, MRE and ARFI quantification were performed on the same day. All elastography methods used in this study are based on quantitative shear wave elastography using a dynamic type of force. An assessment of liver enzymes was not considered, as no significant correlation to hepatic parenchyma damage was observed in several previous studies including a significantly larger number of AATD patients [2,7,23].

### 2.1 MRE

**Fig 1:**
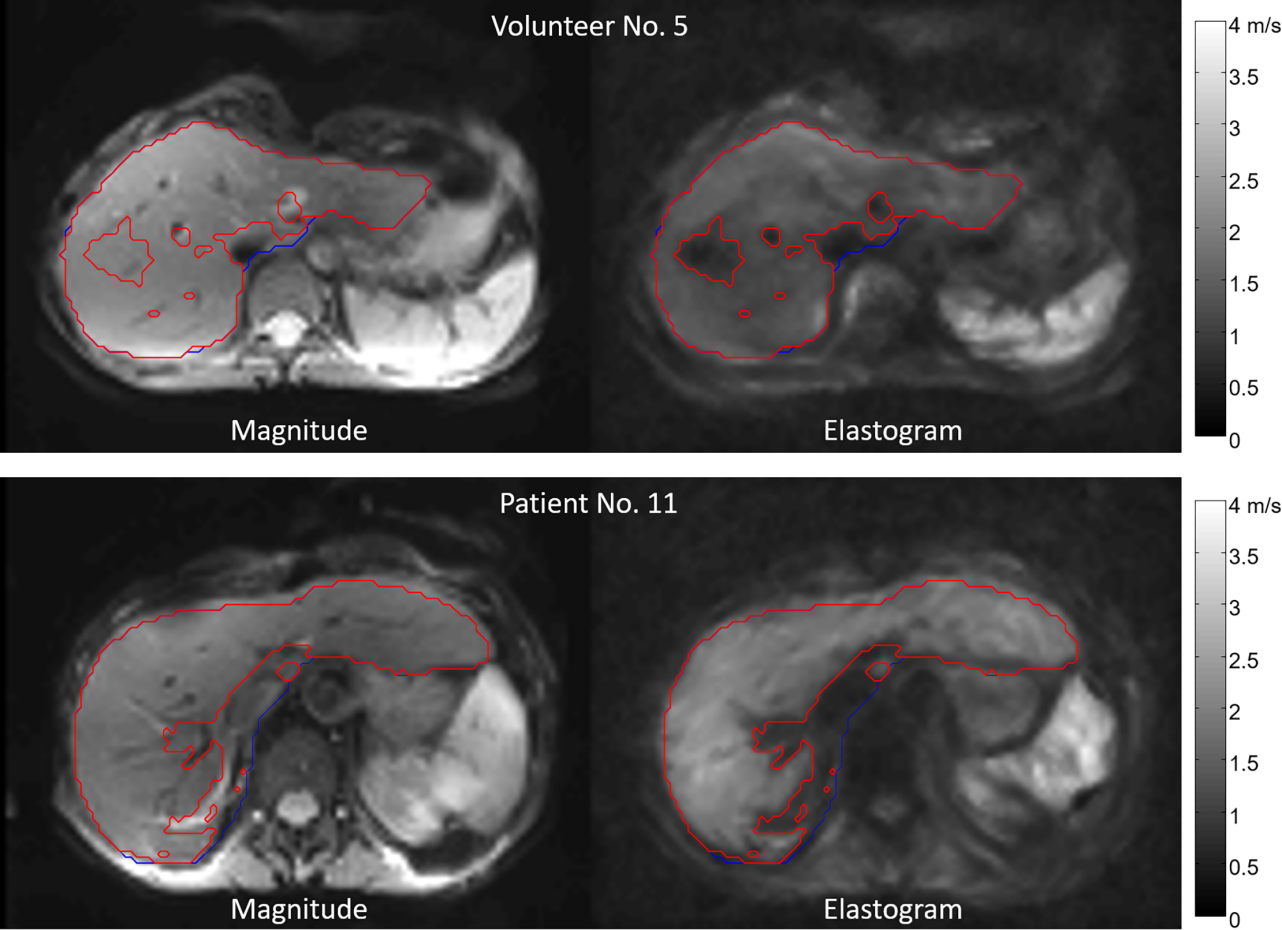
MRE. Axial magnitude image of the liver and corresponding elastogram. ROI for the segmentation of the liver contour (blue) and ROI with a cutoff of 1 m/s (red). Upper row: Healthy volunteer No. 5 with an average SWS of 1.30 ± 0.17 m/s. Bottom row: Patient No. 11. The brighter color in the elastogram indicates an increased SWS of 1.72 ± 0.30 m/s.

Patients and healthy volunteers underwent MRE on a 1.5-Tesla MRI scanner (Magnetom Aera, Siemens Healthcare, Erlangen, Germany) with a 12-channel phased-array surface coil. MRE was performed as described by Hirsch *et al*. 2014 using a piezoelectric driver integrated into the patient table and fast single-shot 3D wave-field acquisition at seven drive frequencies between 30–60 Hz with an increment of 5 Hz [24]. For each frequency, 9 slices, 3×3×5 mm voxel size, 2 averages, and 8 time steps were acquired. MRE image acquisition took approximately 4:30 min and was performed in free and shallow breathing. For postprocessing, Matlab 9.0 R2016a (The Mathworks, Inc., Natick, MA, USA) was used. Elastograms were generated using the *k*-MDEV algorithm [25]. Regions of interest (ROI) were generated in two steps: (i) manual segmentation of the liver contour using the magnitude image and (ii) selective removal of large vessels and other areas with little shear wave excitation using a predefined threshold. The best results were obtained with a threshold of 1 m/s (Fig 1). Fibrosis assessment was classified as reliable after visual evaluation of the magnitude image and the elastogram. For MRE, there are no directly comparable non-invasive cut-off values for fibrosis staging at present due to the use of a newly developed setup with a piezoelectric driver and multifrequency dual parameter reconstruction. Nevertheless, MRE is the best possible non-invasive comparison for USE available to date.

For morphological liver assessment, the following MRI pulse sequences were acquired: axial T1-weighted dual gradient-echo in-phase (IP) and out-of-phase (OP) sequences and axial and coronal T2-weighted half-fourier acquisition single-shot turbo spin echo (HASTE) sequences. Dual gradient-echo signal intensities (SI) were used to calculate liver fat content (LFC in %) according to Fischer *et al*. 2012: LFC = (SI_IP_ ± SI_OP_) / (2SI_IP_) [26]. Negative LFC values were set to zero. A large ROI was selected avoiding vessels and artifacts.

### 2.2 USE

**Fig 2:**
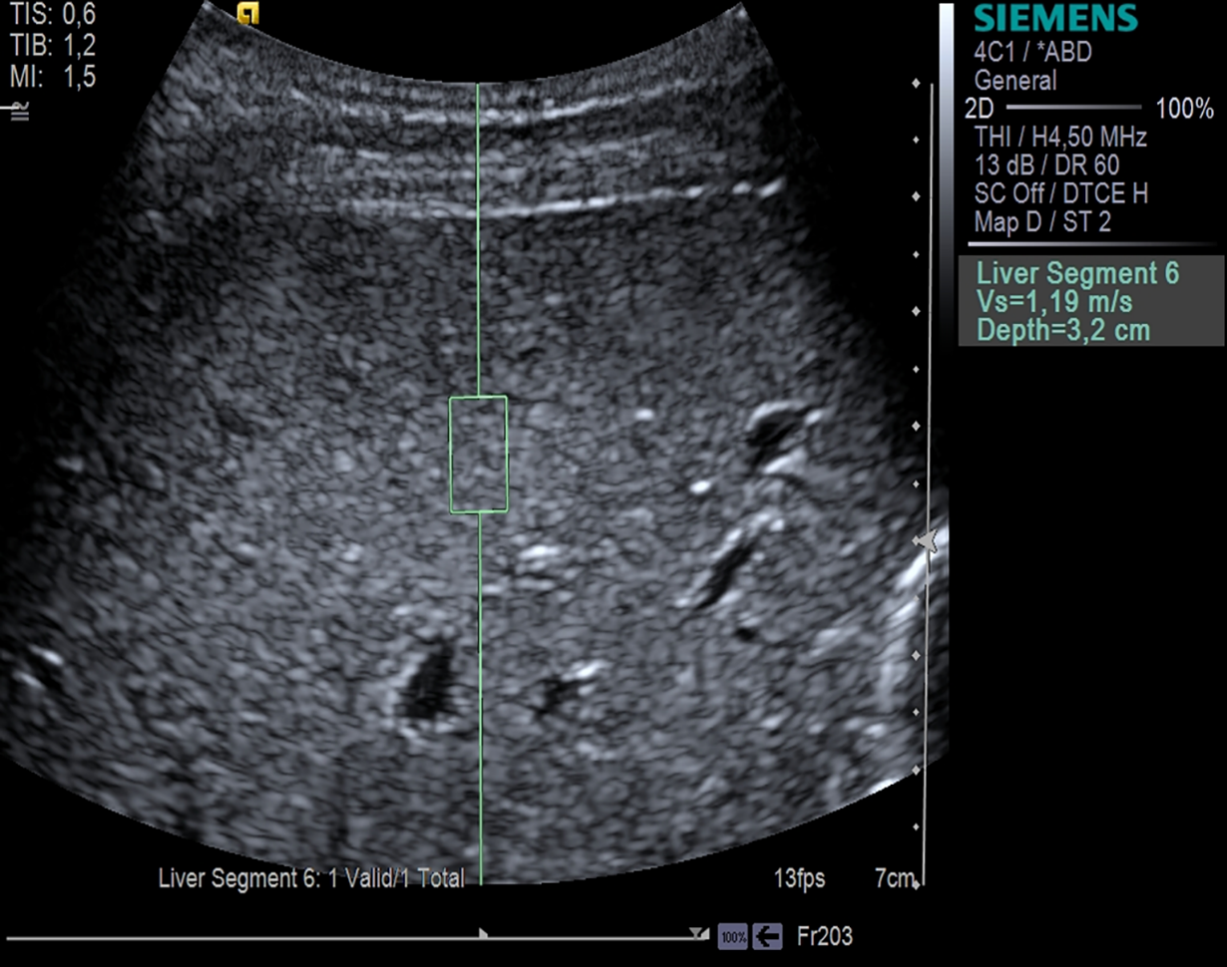
ARFI quantification. Image of a healthy volunteer. In the B-mode image, a rectangular ROI is placed in a homogeneous area in liver segment VI.

**Fig 3:**
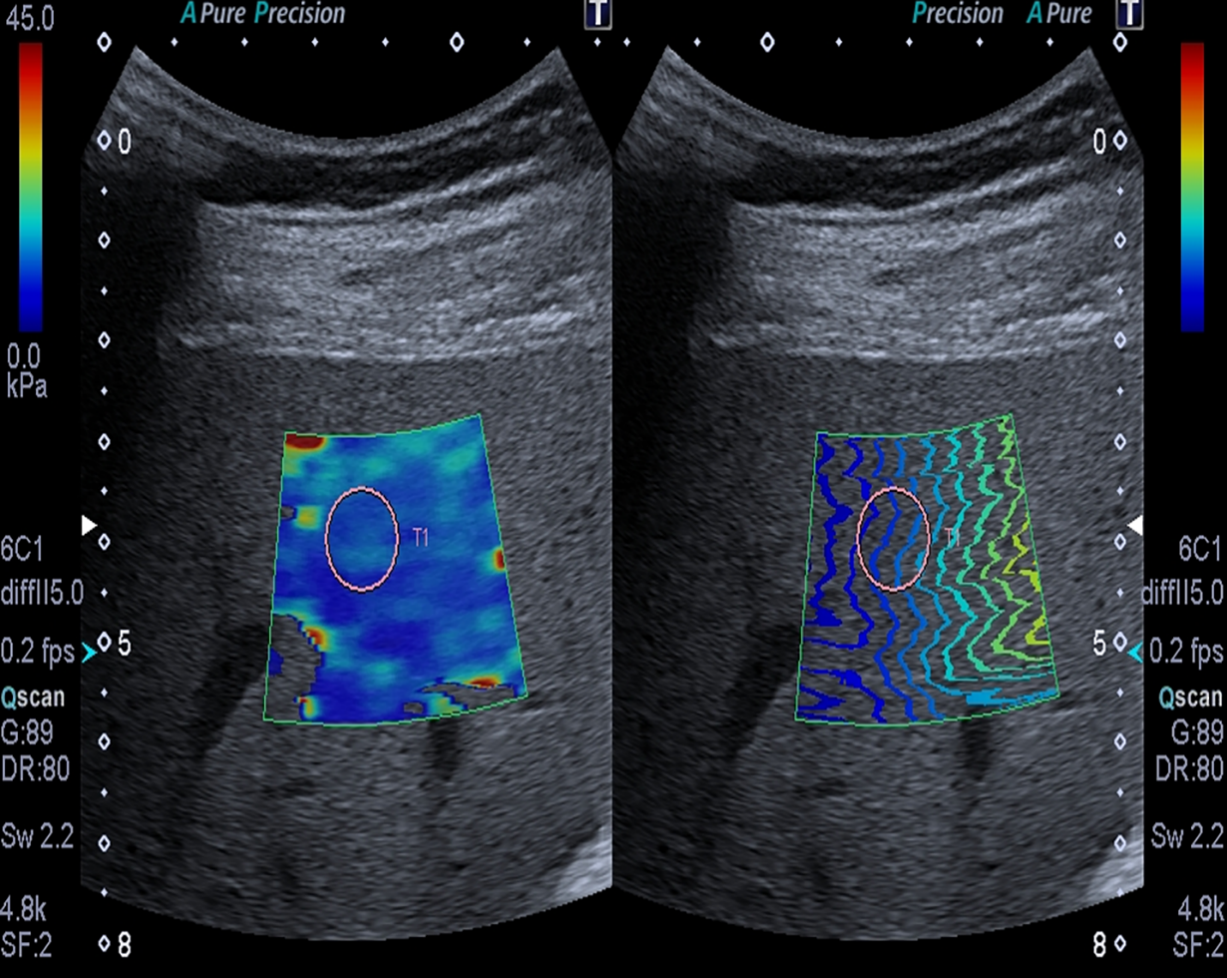
2D-SWE. Images of patient No. 6 with stiffness color map (left) and shear waves (right). A round ROI is placed in liver segment VI in an area of homogeneous color (left) showing an increased SWS of 3.13 ± 0.22 m/s (9.80 ± 1.40 kPa).

Patients and healthy volunteers were examined with ARFI quantification (Virtual Touch Quantification, curved array 4C1, Acuson S2000, Siemens Healthcare, Erlangen, Germany). Additionally, patients were examined with 2D-SWE (curved array 6–1, Aplio 500, Toshiba Medical Systems Corporation, Otawara, Japan). USE was performed according to the guidelines of the European Federation of Societies for Ultrasound in Medicine and Biology (EFSUMB) from 2017 [27]. All USE measurements were performed during breath hold in liver segment VI using an intercostal approach with the subject in supine position and the right arm in abduction. Measurements were performed approximately 2 cm beneath the liver capsule (Figs 2 and 3). For 2D-SWE, a temporal stability of the elastogram of approximately 5 s during breath hold was strived for. Ten measurements were obtained for the calculation of shear wave speed (SWS in m/s). The USE reference values (Table 1) we used make no distinction between stage F0 and F1 fibrosis [20,21]. For comparison of USE and MRE, mean SWS and its standard deviation (SD) were calculated for all methods. An USE assessment was classified as reliable with a success rate of ≥ 60%.

**Table 1:** Reference values.

Fibrosis stage	ARFI quantification	2D-SWE	
	m/s	m/s	kPa
F0-1	≤1.34	≤2.65	≤7.00
F2	≥1.35	≥2.66	≥7.10
F3	≥1.61	≥3.03	≥9.20
F4	≥1.87	≥3.67	≥13.5
	Nierhoff *et al*. 2013	Herrmann *et al*. 2015	

USE reference values for ARFI quantification and 2D-SWE with histological scoring system for fibrosis staging according to METAVIR: F0-1 = no or minimal fibrosis, F2 = significant fibrosis, F3 = severe fibrosis, F4 = cirrhosis [11,20,21]. 2D-SWE reference values were transformed from stiffness (kPa) to SWS (m/s).

### 2.3 Statistical analysis

Statistical significance was assessed using a two-sided t-test and Pearson’s linear correlation coefficient with a level of significance of *P*<0.05.

## 3. Results

**Fig 4:**
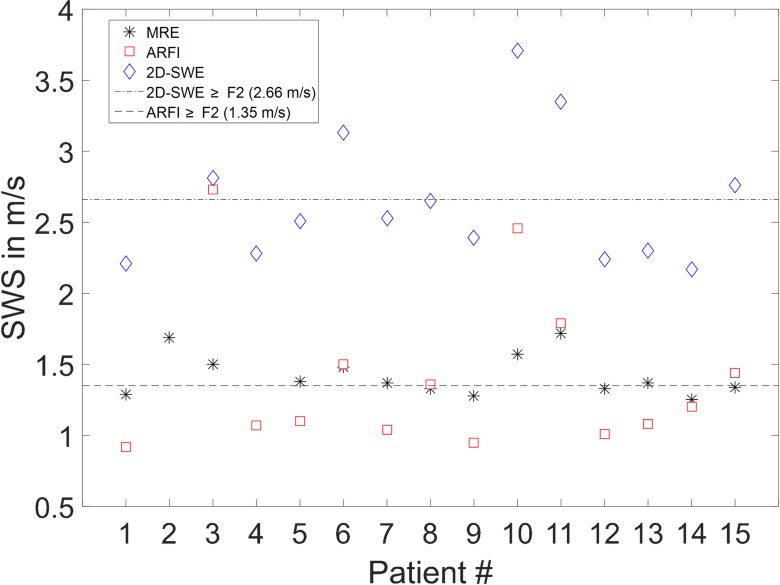
Overview SWS. Plot of SWS determined with all three methods in all patients. Additionally, the reference value for stage F2 fibrosis is shown for ARFI quantification and 2D-SWE.

All parameters determined in patients and healthy volunteers are compiled in Table 2. SWS results obtained by MRE, ARFI quantification, and 2D-SWE in patients are displayed in Fig 4. The percentage of women in the patient and healthy volunteer groups was 47% and 50%, respectively. Patients and healthy volunteers do not show a statistically significant difference for LFC, BMI, and age (*P* = 0.3240, 0.9270, and 0.2690, respectively). Therefore, the two groups were considered suitable for comparison. The mean values and SD of LFC, BMI, and age of patients and healthy volunteers were 5.51 ± 8.06% and 3.10 ± 5.06%, 24.07 ± 3.67 kg/m^2^ and 24.19 ± 3.67 kg/m^2^, and 56 ± 10 years and 52 ± 12 years, respectively. Mean LFC is low in both groups. Patient No. 1 and healthy volunteer No. 7 had a BMI > 30 kg/m^2^ (31.89 kg/m^2^ and 31.80 kg/m^2^, respectively). All other participants showed a BMI < 30 kg/m^2^. According to the above-mentioned reliability criteria, measurements failed for patient No. 2 (ARFI quantification, 2D-SWE), and patient No. 4 (MRE). Therefore, both patients were excluded from pairwise comparison of all parameters in the correlation analysis.

**Table 2:** Compilation of results obtained in all patients and healthy volunteers.

Pat. #	Genotype	Sex m/f	Age years	BMI kg/m^2^	LFC %	MRE m/s	ARFI m/s	2D-SWE m/s
1	PiZZ	f	45	31.89	0.00	1.29 (0.17)	0.92 (0.17)	2.21 (0.25)
2	PiZZ	m	56	21.46	4.89	1.69 (0.36)	-	-
3	PiZZ	m	56	26.59	0.00	1.50 (0.27)	2.73 (1.46)	2.81 (0.22)
4	PiMZ	m	52	25.98	0.00	-	1.07 (0.16)	2.28 (0.22)
5	PiZZ	m	62	20.96	5.48	1.38 (0.24)	1.10 (0.17)	2.51 (0.26)
6	PiZZ	m	53	22.72	16.30	1.48 (0.23)	1.50 (0.27)	3.13 (0.22)
7	PiZZ	f	56	24.80	0.00	1.37 (0.20)	1.04 (0.14)	2.53 (0.32)
8	PiZZ	m	52	26.26	28.29	1.33 (0.19)	1.36 (0.41)	2.65 (0.23)
9	PiZZ	f	64	28.52	10.95	1.28 (0.18)	0.95 (0.13)	2.39 (0.25)
10	PiZZ	m	59	23.89	0.00	1.57 (0.32)	2.46 (0.99)	3.71 (0.80)
11	PiZZ	f	72	18.07	1.01	1.72 (0.30)	1.79 (0.21)	3.35 (0.33)
12	PiMZ	f	44	21.61	0.00	1.33 (0.18)	1.01 (0.08)	2.24 (0.27)
13	PiMZ	f	65	23.11	7.47	1.37 (0.15)	1.08 (0.19)	2.30 (0.20)
14	PiMZ	f	37	18.91	0.00	1.25 (0.14)	1.20 (0.05)	2.17 (0.30)
15	PiZZ	m	71	26.26	8.21	1.34 (0.18)	1.44 (0.12)	2.76 (0.18)
Mean			56	24.07	5.51	1.42 (0.22)	1.40 (0.33)	2.65 (0.29)
SD			10	3.67	8.06	0.15	0.56	0.47
	Vol. #	Sex m/f	Age years	BMI kg/m^2^	LFC %	MRE m/s	ARFI m/s	
	1	m	42	22.46	0.00	1.36 (0.22)	1.07 (0.04)	
	2	f	62	19.38	0.00	1.35 (0.22)	1.23 (0.05)	
	3	m	31	23.52	0.00	1.38 (0.22)	1.10 (0.04)	
	4	m	57	26.23	5.14	1.34 (0.19)	0.72 (0.03)	
	5	f	37	23.18	3.94	1.30 (0.17)	1.24 (0.09)	
	6	m	64	29.38	17.01	1.42 (0.16)	0.87 (0.07)	
	7	f	48	31.80	7.89	1.30 (0.18)	0.92 (0.06)	
	8	f	60	24.68	4.40	1.32 (0.25)	1.11 (0.04)	
	9	m	37	21.60	0.00	1.44 (0.30)	1.33 (0.05)	
	10	f	51	19.15	0.00	1.29 (0.16)	1.21 (0.06)	
	11	m	75	26.26	0.00	1.35 (0.24)	0.97 (0.09)	
	12	m	57	25.08	0.00	1.34 (0.20)	1.39 (0.12)	
	13	f	61	19.72	0.00	1.49 (0.22)	1.03 (0.04)	
	14	f	47	23.24	0.00	1.27 (0.15)	1.04 (0.04)	
	15	m	59	22.31	0.00	1.23 (0.18)	1.07 (0.05)	
	16	f	41	29.06	11.19	1.32 (0.19)	1.25 (0.20)	
	Mean		52	24.19	3.10	1.34 (0.20)	1.10 (0.07)	
	SD		12	3.67	5.06	0.07	0.18	

Numbers in parentheses are measured standard deviations. Lacking numbers refer to failed measurements according to defined reliability criteria. Pat: patient. Vol: healthy volunteer. f: female. m: male. BMI: Body Mass Index. LFC: Liver Fat Content calculated from T1-weighted dual gradient-echo sequence [26]. MRE: Magnetic Resonance Elastography. ARFI: Acoustic Radiation Force Impulse Quantification. 2D-SWE: 2D-Shear Wave Elastography.

### 3.1 MRE

Mean SWS tended to be higher in patients compared to healthy volunteers with 1.42 ± 0.15 m/s and 1.34 ± 0.07 m/s, respectively. However, no statistical significance was reached in this small cohort (*P* = 0.0696). Despite the concerns of some patients having to cough due to AATD-related pulmonary emphysema during the MRE examination, there were no significant limitations in image quality due to motion artifacts, except for patient No. 4.

### 3.2 ARFI quantification

Mean SWS was significantly higher in patients compared to healthy volunteers with 1.40 ± 0.56 m/s and 1.10 ± 0.18 m/s, respectively (*P* = 0.0475). According to the reference values from the meta-analysis of Nierhoff *et al*. 2013, the median fibrosis stage was F2 (F0-1: *n* = 8, F2: *n* = 3, F3: *n* = 1, F4: *n* = 2) in patients and F0-1 in volunteers (Table 1) [20]. A single healthy volunteer (No. 12) showed an increased SWS of 1.39 ± 0.12 m/s but no increased MRE value compared to all other healthy volunteers and no history of liver disease. Therefore, this result may be considered an outlier possibly caused by eating shortly before the USE examination.

### 3.3 2D-SWE

Mean SWS was 2.65 ± 0.47 m/s in patients. According to the reference values from the meta-analysis of Herrmann *et al*. 2015, the median fibrosis stage was F0-1 (F0-1: *n* = 9, F2: *n* = 2, F3: *n* = 2, F4: *n* = 1) with a tendency toward F2 (F2≥2.66 m/s) (Table 1) [21].

### 3.4 Parameter correlation

**Fig 5:**
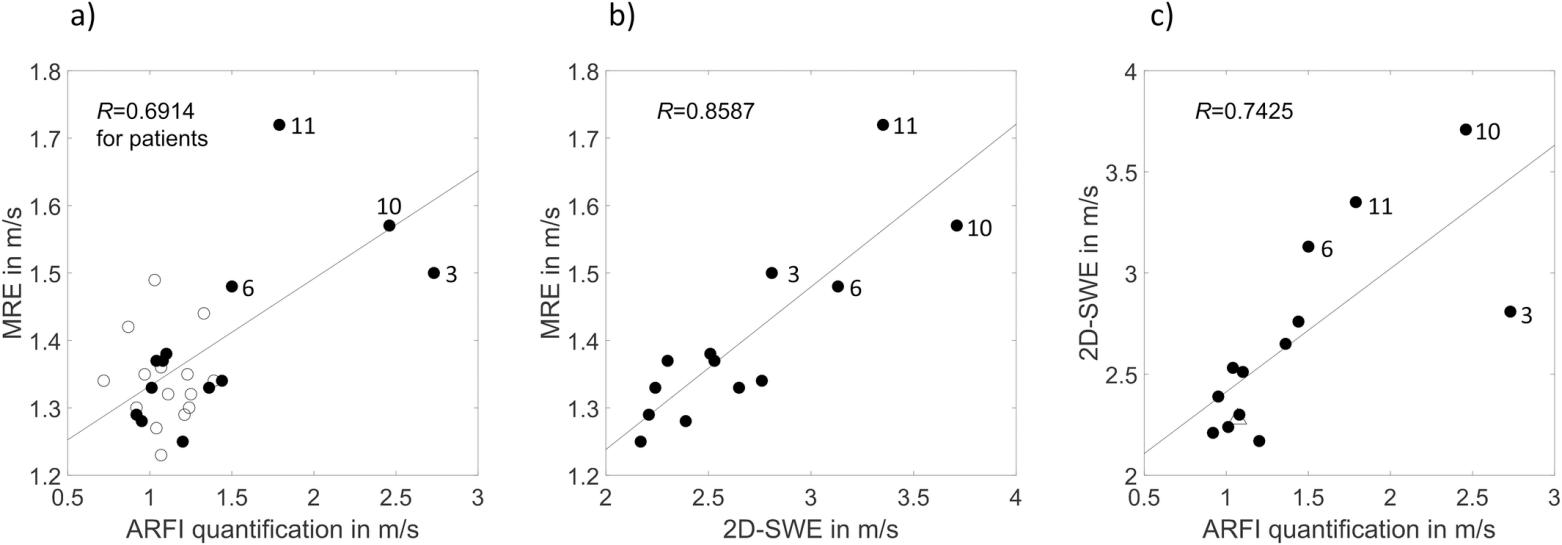
Parameter correlation. a)-c) Linear correlation of MRE, ARFI quantification, and 2D-SWE in patients (●). All *P*-values ≤ 0.0089. Patients with the highest SWS are indicated by numbers: patient Nos. 3, 6, 10, and 11. Based on USE reference values, these patients have fibrosis ranging from F2 to F4 [20,21]. a) Additionally, correlation of ARFI quantification/MRE is shown for healthy volunteers (ᴏ). c) Patient No. 4 without MRE data is indicated by (△).

The results of pairwise comparison of all parameters for patients (*n* = 13) in whom all three methods were completed successfully are presented in Table 3. Linear correlation of MRE, ARFI quantification, and 2D-SWE is displayed in Fig 5. A high correlation is evident for the different elastography methods: 2D-SWE/MRE, ARFI quantification/2D-SWE, and ARFI quantification/MRE (*R* = 0.8587, 0.7425, and 0.6914; *P*≤0.0089). The correlation of ARFI quantification/MRE is stronger for patients alone than for patients and healthy volunteers taken together (*R* = 0.6914 and 0.6108, respectively; *P*≤0.0089). No statistically significant influence of LFC, BMI, and age on the SWS of MRE, ARFI quantification, and 2D-SWE is evident (Table 3).

**Table 3:** Parameter correlation.

	MRE	ARFI	2D-SWE	LFC	BMI	Age
MRE	-	0.6914*	0.8587*	-0.1873	-0.4013	0.4972
ARFI	0.0089	-	0.7425*	-0.1693	-0.0794	0.2086
2D-SWE	0.0002	0.0036	-	0.0279	-0.2208	0.4571
LFC	0.5401	0.5803	0.9280	-	0.1829	0.1091
BMI	0.1741	0.7964	0.4685	0.5498	-	-0.0391
Age	0.0839	0.4941	0.1163	0.7228	0.8990	-

Pairwise comparison of parameters obtained in patients (*n* = 13) with linear correlation coefficients *R* (upper triangle array) and *P*-values (bottom triangle array). Only patients with valid measurements obtained with all three elastography methods were considered. *R*-values marked with an asterisk (*) indicate a level of significance of *P*<0.05.

## 4. Discussion

To our knowledge, this is the first study investigating the non-invasive assessment of AATD-related liver fibrosis using both USE (ARFI quantification and 2D-SWE) and MRE in the same patients and volunteers. Additionally, this is the first study investigating 2D-SWE in the assessment of AATD-related liver fibrosis. At the time the examinations were performed, all AATD patients were asymptomatic and none had a clinical indication for liver biopsy. However, this asymptomatic course of liver disease, which is typical for AATD, frequently leads to a clinically mute progression to cirrhosis and HCC and delays the diagnosis [5–7]. In view of this situation, there is a need for a non-invasive and precise diagnostic method for detecting and characterizing AATD-related liver fibrosis.

Two groups emerge when the results in patients and healthy volunteers are taken together. Group 1: Most patients and healthy volunteers have similarly low SWS determined by MRE and ARFI quantification. This group is classified as normal. In comparison to MRE, ARFI quantification yields a wider range of SWS values. Possibly, this might explain the fact that MRE could only show a tendency but not statistical significance for higher SWS in patients in comparison to healthy volunteers. Group 2: Four AATD patients have consistently higher SWS with all three diagnostic methods—MRE, ARFI quantification, and 2D-SWE. Therefore, this group is classified as pathological.

The higher correlation of ARFI quantification/MRE in patients alone versus patients and healthy volunteers taken together might possibly be explained by a better diagnostic accuracy in classifying more advanced fibrosis stages, as demonstrated in a previous study by Asbach *et al*. [15]. Mostafavi *et al*. assessed 47 AATD patients (32 PiZZ, 15 PiSZ) using ARFI quantification. They found median liver stiffness to be significantly increased in PiZZ and PiSZ men compared to PiMM men [28]. In agreement with our study, no relationship between LFC and ARFI quantification was evident. Conversely, Mostafavi *et al*. showed a statistically significant correlation between BMI and ARFI quantification. This discrepancy to our findings might be attributable to the investigation of different study populations. Median BMI was lower in our patient group than in the group studied by Mostafavi *et al*. (23.9 kg/m^2^, range: 18.1–31.9 kg/m^2^ versus 24.0–26.5 kg/m^2^, range: 19.0–43.0 kg/m^2^). In our study, only one patient had a BMI > 30 kg/m^2^. Kim *et al*. investigated 9 PiZZ patients using MRE and correlated the findings with liver biopsy [29]. Four patients in this study had histologically proven fibrosis (F1: *n* = 2, F2 or higher: *n* = 2). Therefore, they defined a cutoff of 1.73 m/s (3 kPa) for the detection of any fibrosis (F≥1). However, Kim *et al*. used a different technical setup, and, therefore, this reference value is not applicable to our study. The high correlation of USE methods in our study is in agreement with a study of Gerber *et al*. including 132 patients with various chronic liver diseases such as chronic hepatitis B and C, steatohepatitis, and autoimmune hepatitis. It was shown that ARFI quantification and 2D-SWE yield equally good results in the non-invasive assessment of liver fibrosis [30]. However, the focus of our study was to investigate and compare MRE and USE in patients with a different and as yet rather poorly understood liver disease.

Limitations of this study include the missing histopathological gold standard, the small number of patients, due to the diagnostic challenge posed by AATD, the time interval between USE and MRE, and the fact that 2D-SWE was only performed in patients and not in healthy volunteers. Moreover, SWS of most patients indicated fibrosis in the F0-1 range. Therefore, the high correlation of different methods is based on a few patients with elevated SWS. Despite the long time interval between USE and MRE for patients, we presume, based on a previous study by Singh *et al*., a rather slow disease progression and, hence, only a small dynamic of liver fibrosis and its viscoelastic properties [31].

## 5. Conclusion

The high correlation and consistent identification of patients with pathologically increased SWS using MRE, ARFI quantification, and 2D-SWE suggest that elastography has the potential to become a suitable imaging tool for the assessment of AATD-related liver fibrosis. It seems possible that AATD-related liver fibrosis can be detected early and before the development of cirrhosis. These promising results provide motivation for further investigation of non-invasive assessment of AATD-related liver fibrosis using elastography.
